# Adrenomedullin Deficiency and Aging Exacerbate Ischemic White Matter Injury after Prolonged Cerebral Hypoperfusion in Mice

**DOI:** 10.1155/2014/861632

**Published:** 2014-06-16

**Authors:** Yumiko Mitome-Mishima, Nobukazu Miyamoto, Ryota Tanaka, Tatsuo Shimosawa, Hidenori Oishi, Hajime Arai, Nobutaka Hattori, Takao Urabe

**Affiliations:** ^1^Department of Neurosurgery, Juntendo University School of Medicine, Tokyo, Japan; ^2^Department of Neurology, Juntendo University Urayasu Hospital, 2-1-1 Tomioka, Urayasu, Chiba 279-0021, Japan; ^3^Department of Neurology, Juntendo University School of Medicine, Tokyo, Japan; ^4^Department of Clinical Laboratory, Faculty of Medicine, The University of Tokyo, Tokyo, Japan; ^5^Department of Neuroendovascular Therapy, Juntendo University School of Medicine, Tokyo, Japan

## Abstract

Adrenomedullin was originally isolated from pheochromocytoma cells and reduces insulin resistance by decreasing oxidative stress. White matter lesions induced by aging and hyperglycemia play a crucial role in cognitive impairment in poststroke patients. Here, we examine whether adrenomedullin deficiency and aging exacerbate ischemic white matter injury after prolonged cerebral hypoperfusion. Adrenomedullin heterozygous, wild-type young/aged mice were subjected to prolonged hypoperfusion. Prolonged cerebral hypoperfusion followed by immunohistochemical analysis was used to evaluate white matter injury. After prolonged hypoperfusion, white matter damage progressed in a time-dependent manner in AM^+/−^ group compared with the wild-type group. The number of oligodendrocyte progenitor cells gradually increased after prolonged hypoperfusion, whereas oligodendrocytes decreased following a transient increase, but the ratio of increase was mild in the AM^+/−^ group (*P* < 0.05). Oxidative stress was detected in oligodendrocytes, with a larger increase in the AM^+/−^ group (*P* < 0.05). Aged mice showed the same tendency, but white matter damage was worse, especially in the aged AM^+/−^ group. Our results demonstrated that white matter injury was increased in adrenomedullin deficiency, which induced oxidative stress. White matter injury was more exacerbated because of hyperglycemia in aged AM^+/−^ group. Adrenomedullin may be an important target in the control of ischemic white matter injury.

## 1. Introduction

Adrenomedullin (AM) is a hypotensive peptide discovered in human pheochromocytoma [1]. AM is mainly produced in and secreted by vascular endothelial cells [2], but it is also secreted in various organs including the heart, lung, kidney, adipose tissues, and central nervous system [3]. AM has a variety of effects on the vasculature that include vasodilation, regulation of permeability, inhibition of endothelial cell apoptosis and oxidative stress, regulation of smooth muscle cell proliferation, and promotion of angiogenesis [1, 4]. The vasodilator action of AM is evident from its ability to elevate intracellular cyclic AMP (cAMP) in vascular smooth muscle cells [5, 6].

AM^+/−^ mice showed that AM has the potential not only to decrease blood pressure, but also to protect organs from damage [7]. Some studies have demonstrated that oxidative stress in vascular endothelial and smooth muscle cells can increase production of AM, which acts as an endogenous antioxidant to protect organs [8–10]. Moreover, AM^+/−^ mice accumulate higher oxidative stress and insulin resistance in aging compared with wild-type (WT) mice [11]. Age-related accumulation of oxidative stress is involved in blood pressure regulation and insulin resistance in aged AM^+/−^ mice, and AM is thus an endogenous substance counteracting oxidative stress-induced insulin resistance associated with aging. For that reason, aged AM^+/−^ mice develop hyperglycemia [11].

In the central nervous system, in which AM is mainly expressed in neurons and the endothelium [12], it is reported that transient ischemia boosts AM expression for >15 days after ischemia [13]. A recent study demonstrated that circulating AM is a highly potent and effective modality for restoring perfusion, promoting arteriogenesis and angiogenesis in the chronically ischemic brain, inhibiting oxidative damage in cerebral microvessels, preserving ischemic white matter (WM) integrity, and attenuating working memory deficits in a mouse model of subcortical vascular dementia [14]. In the present study, we first used WT mice and an AM^+/−^ mouse model of prolonged cerebral hypoperfusion to examine the effect of AM on ischemic white matter injury in the paramedian part of the corpus callosum, in which white matter lesions are the most intense [15]. Next, we compared white matter injury formation in aged WT and AM^+/−^ mice to evaluate the effect of high blood sugar and aging on prolonged cerebral hypoperfusion.

## 2. Experimental Procedures

### 2.1. Experimental Protocol

All animal procedures were approved by the Animal Care Committee of Juntendo University. Twelve-week-old and 15-month-old male AM^+/−^ mice with a disruption in the AM peptide (*n* = 58) and C57BL/6 WT mice (*n* = 68) were used in this study. Mice were maintained on a 12 h light/dark cycle with free access to food and water. AM-knockout homozygotes die in utero for unknown reasons; therefore, we examined AM^+/−^ mice, in which the serum and organ concentrations of AM are about 50% of those reported in WT mice [7, 8].

Prolonged cerebral hypoperfusion was induced by bilateral common carotid artery stenosis (BCAS) using microcoils made of piano wire (wire diameter of 0.08 mm) with an inner diameter of 0.18 mm, a pitch of 0.50 mm, and a total length of 2.5 mm (Samini Co., Ltd., Shizuoka, Japan), as previously described [16]. Briefly, the animals were anesthetized with 1.5–2.0% isoflurane in 30% oxygen and 70% nitrogen using a small-animal anesthesia system. Then, both common carotid arteries (CCAs) were exposed and freed from their sheaths via a midline cervical incision. Two 4-0 silk sutures were placed around the distal and proximal parts of the right CCA. Then, the artery was gently lifted by these sutures and placed between the loops of the microcoil just below the carotid bifurcation. The microcoil was twined by rotating it around the CCA. Another microcoil of the same size was twined around the left CCA. Cessation of CBF for more than 1 min was avoided. All procedures for BCAS were accomplished within 15 min. The body temperature was kept at 37.0 ± 0.5°C (mean ± SD) using a heating pad (Unique Medical, Tokyo, Japan) throughout the surgical procedure. Mice were sacrificed before the procedure (preoperation) or at 3, 7, 14, or 28 days after the BCAS (*n* = 5 for each group) (only AM^+/−^ age group, preoperation, or at 14, 28 days; *n* = 3 for each group). Cerebral blood flow (CBF) was recorded through a left temporal window by laser Doppler flowmetry (Omega Wave, Tokyo, Japan) before and after the BCAS, as well as before sacrifice. Plasma glucose level was measured using a blood glucose meter (Johnson & Johnson, New Brunswick, NJ, USA). In this experiment, blood (200 *μ*L) was collected from the ophthalmic venous plexus before the procedure (preoperation) and at 3, 7, 14, and 28 days after the BCAS. At sacrifice, the mice were deeply anesthetized with intraperitoneal injection of 50 mg/kg of pentobarbital followed by transcardial perfusion. The brain was removed immediately* en bloc* and postfixed for 48 h in 4% paraformaldehyde in phosphate-buffered saline (PBS) at 4°C, then immersed in 30% sucrose, and finally cryopreserved until use. The brain was thawed and cut into 20 *μ*m thick consecutive coronal sections of the white matter using a cryostat (CM 1900; Leica Instruments, Nussloch, Germany). The prepared sections were used for histochemical analysis, immunohistochemical analysis, and double immunofluorescence histochemistry.

### 2.2. Histochemical Evaluation of White Matter Lesions

Myelin was stained with Luxol fast blue (Kluver-Barrera (KB) [17]); the degree of white matter change was assessed using a scoring system described in detail previously [18]: grade 0, no observable deficit (normal); grade 1, disarrangement of nerve fibers; grade 2, formation of marked vacuoles; grade 3, disappearance of myelinated fibers.

### 2.3. Immunohistochemistry

After wash in PBS and incubation in 3% H_2_O_2_ followed by washing in PBS and blocking in 10% normal goat serum (Dako Corporation, Carpinteria, CA, USA) in PBS, the sections were immunostained overnight at 4°C with antibodies against anti-GST*π* (dilution, 1 : 100; Medical & Biological Laboratories Co., Nagoya, Japan), anti-PDGFR*α* (dilution, 1 : 100; Santa Cruz Biotechnology, Santa Cruz, CA, USA), ionized calcium-binding adapter molecule 1 (Iba-1, dilution, 1 : 500; Wako Pure Chemicals, Osaka, Japan), anti-pCREB (dilution, 1 : 100; Upstate Biotechnology, Lake Placid, NY, USA), inducible nitric oxide synthase (iNOS, dilution, 1 : 100; BD Biosciences, Tokyo, Japan), anti-8-Hydroxy-deoxyguanosine (8OHdG, dilution, 1 : 25; Japan Institute for the Control of Aging, Shizuoka, Japan), or anti-4-hydroxy hexenal (4-HHE, dilution, 1 : 100; NOF Corporation, Tokyo, Japan). The sections were then washedwith PBS and treated with appropriate secondary antibodies (Vector Laboratories, Burlingame, CA, USA). After washing in PBS, immunoreactivity was visualized by the avidin-biotin complex method (Vectastain ABC kit, Vector Laboratories) and washed in PBS and developed with diaminobenzidine.

### 2.4. Immunofluorescence Histochemistry

Brain sections were washed with PBS and incubated in a blocking solution, 2% Block Ace (Yukijirushi, Sapporo, Japan) in PBS, for 30 min at room temperature. Immunofluorescence staining was performed by incubation of sections overnight with anti-pCREB (dilution, 1 : 100; Upstate Biotechnology) at 4°C. The primary antibodies were detected by incubation with fluorescein isothiocyanate-conjugated secondary antibody (1 : 500; Jackson Immunoresearch Laboratories, West Grove, PA, USA) for 90 min at room temperature. The sections were washed with PBS and mounted on microslide glass with Vectorshield Mounting Medium (Vector Laboratories).

### 2.5. Double Immunofluorescence Histochemistry

Double immunofluorescence histochemical staining was performed to determine the origin and localization of 8OHdG- and HHE-positive cells. Brain sections were washed with PBS and incubated in a blocking solution, 2% Block Ace (Yukijirushi, Sapporo, Japan) in PBS, for 30 min at room temperature. Double immunofluorescence staining was performed by simultaneous incubation of sections overnight with anti-8OHdG (dilution, 1 : 25; Japan Institute for the Control of Aging), anti-4-hydroxy hexenal (4-HHE, dilution, 1 : 100; NOF Corporation), anti-GST*π* (dilution, 1 : 25; Santa Cruz Biotechnology), and anti-PDGFR*α* (dilution, 1 : 200; Abcam, Cambridge, MA, USA) at 4°C. For double labeling, the primary antibodies were detected by incubation with Cy3- or fluorescein isothiocyanate-conjugated secondary antibody (1 : 500; Jackson Immunoresearch Laboratories, West Grove, PA, USA) for 90 min at room temperature. The sections were washed with PBS and mounted on microslide glass with Vectorshield Mounting Medium (Vector Laboratories).

### 2.6. Western Blotting

Mice of each group were decapitated at, before, and after 14 or 28 days of ischemia/reperfusion (*n* = 3 for each group). Samples were taken from the ischemic region (comprising the cortex and striatum on the bilateral side). Aliquots containing 20 *μ*g of protein were subjected to 12.5% sodium dodecyl sulfate-polyacrylamide gel electrophoresis. The protein bands were transferred onto polyvinylidene fluoride membrane (Millipore). The membranes were blocked with 1% bovine serum albumin (BSA) in 0.05% Tween 20 and sequentially incubated with the primary antibodies anti-MBP (dilution, 1 : 5,000; Abcam). After incubation with the appropriate horseradish peroxidase-conjugated secondary antibody (dilution, 1 : 5,000; Amersham Life Science, Buckinghamshire, UK) for 1 h at room temperature, the immunoreactive bands were visualized in the linear range with enhanced chemiluminescence ECL Prime Western Blotting Detection (Amersham Biosciences, Piscataway, NJ, USA). The western blots were evaluated quantitatively using a computerized digital image system (LAS-4000 mini; GE Healthcare, Tokyo, Japan). Equal protein loading was confirmed by measuring *α*-tubulin. The Oxyblot protein oxidation detection kit (Chemicon) was used following the manufacturer's instructions.

### 2.7. Cell Counts and Statistical Analysis

**Figure 1:**
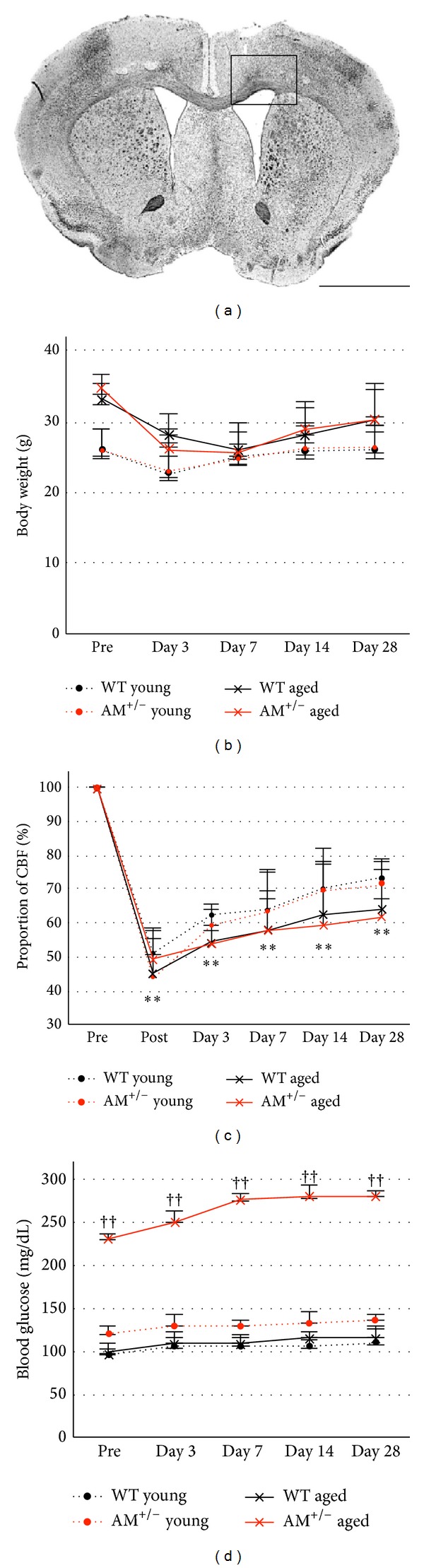
Physiological parameters after prolonged cerebral hypoperfusion. (a) Schematic representation of the lateral portions of the corpus callosum (enclosure). (b) Temporal changes in body weight before BCAS (pre) and at days 3, 7, 14, and 28 after BCAS. (c) Proportion of cerebral blood flow (CBF) before BCAS (pre), immediately after BCAS (post), and at days 3, 7, 14, and 28 after BCAS. (d) Temporal changes in blood glucose measured before BCAS (pre) and at days 3, 7, 14, and 28 after BCAS. Data are mean ± SEM of five mice in each group. *P* < 0.05, **P < 0.001, compared with the preoperation group (pre). *P* < 0.05, ^††^ *P* < 0.001, compared with the same time points. Pre: preoperation.

In the double immunofluorescence histochemical analysis, positively stained cells in the white matter (lateral portions of the corpus callosum) were counted using three sections per animal (Figure 1(a): 0.25 mm^2^, bregma +1.18 mm, +0.98 mm, and +0.74 mm). All experiments and measurements (including cell count) were performed in a blinded and randomized manner. Power estimates were calculated based on *α* = 0.05 and *β* = 0.8 to obtain group sizes appropriate for detecting effect sizes in the range of 30–50% for in vivo models. Statistical significance was evaluated using the unpaired* t*-test to compare differences between the two groups and a one-way analysis of variance followed by Tukey's honestly significant difference test for multiple comparisons. Data are expressed as mean ± standard deviation. A *P* value of <0.05 was considered statistically significant.

## 3. Results and Discussion

### 3.1. Changes in the Physiological Parameter

Prolonged cerebral hypoperfusion-induced WM lesions contribute to the development of cognitive impairments that are common and difficult to cure in the elderly population. A simple and reproducible mouse model of prolonged cerebral hypoperfusion is needed for investigation of the neurochemical and molecular mechanisms of WM lesion development. In 2004, Shibata et al. [16] reported a novel mouse model of prolonged cerebral hypoperfusion. Moreover, another study showed that animal categorization according to CBF value led to more definite and reproducible WM lesions than in previous studies [15]. This model was exclusively suitable for C57BL/6 mice. In our study, AM^+/−^ and WT mice were subjected to BCAS using microcoils with a diameter of 0.18 mm.

Physiological parameter is presented in Figure 1. The body weight decreased after the surgery but tended to recover to the baseline until day 28 in all groups (Figure 1(b)). Although the mice had a lower body weight after operation, no significant difference was noted at any postoperative interval.  Figure 1(c) plots the mean CBF values, indicating a decrease of about 50% immediately after the occlusion. On day 3, the CBF values began to recover but remained significantly lower in all groups until 28 days, as compared with the preoperation measures (*P* < 0.001). There were no intergroup differences in CBF values among each group.

### 3.2. Changes in the White Matter Lesions and the Number of Oligodendrocyte Linage Cells after Prolonged Cerebral Hypoperfusion between Young Groups

**Figure 2:**
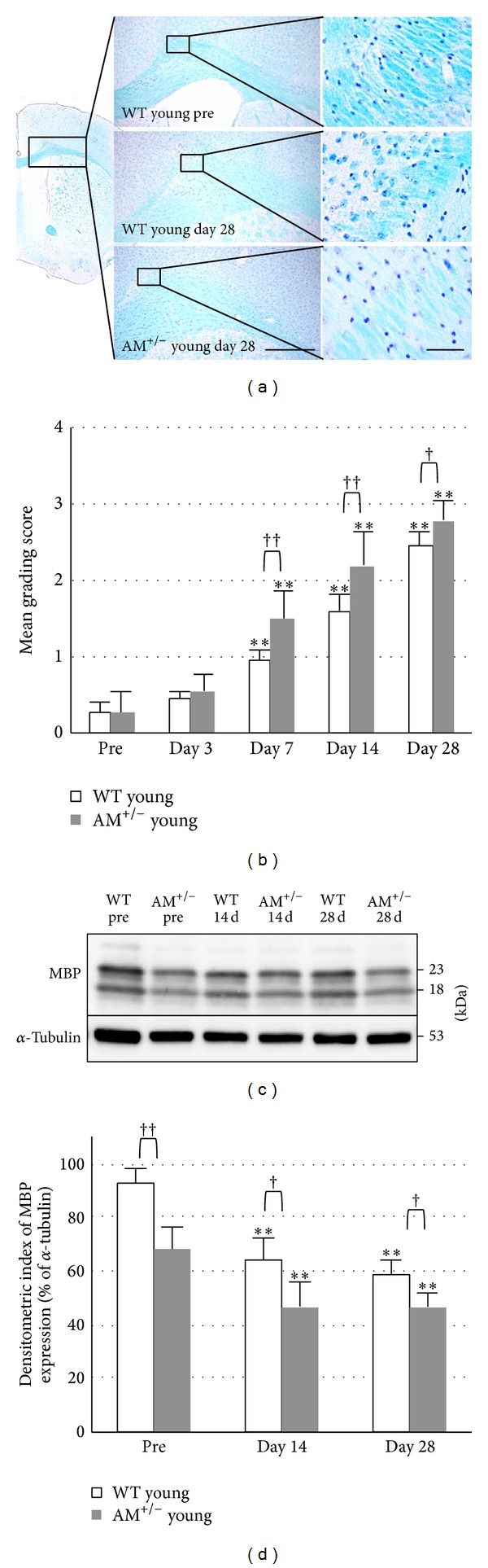
Serial changes in white matter lesion after prolonged cerebral hypoperfusion in young groups. (a) Photomicrographs of KB staining in the corpus callosum before BCAS (pre) and at day 28 after BCAS. Scale bars = 200 *μ*m (low magnification), 20 *μ*m (high magnification). (b) Mean grading score of KB staining. ((c), (d)) Western blotting (c) and densitometric analysis (d) of MBP. Data are mean ± SEM of five mice in each group. *P* < 0.05, **P < 0.001, compared with the preoperation group (pre). ^†^ *P* < 0.05, ^††^ *P* < 0.001, compared with the same time points. Pre: preoperation.

All mice were confirmed to have developed white matter lesion after prolonged cerebral hypoperfusion. The white matter lesion and grading scores are summarized in Figures 2(a) and 2(b). The lesions were not detected until 7 days after BCAS. After 14 days, the WM lesions were evaluated as grade 1 or 2 and after 28 days, severe rarefaction occurred in these regions. Western blot analysis also demonstrated that the density of the MBP-positive band (18, 23 kDa) decreased in a time-dependent manner (*P* < 0.05, Figures 2(c) and 2(d)).

**Figure 3:**
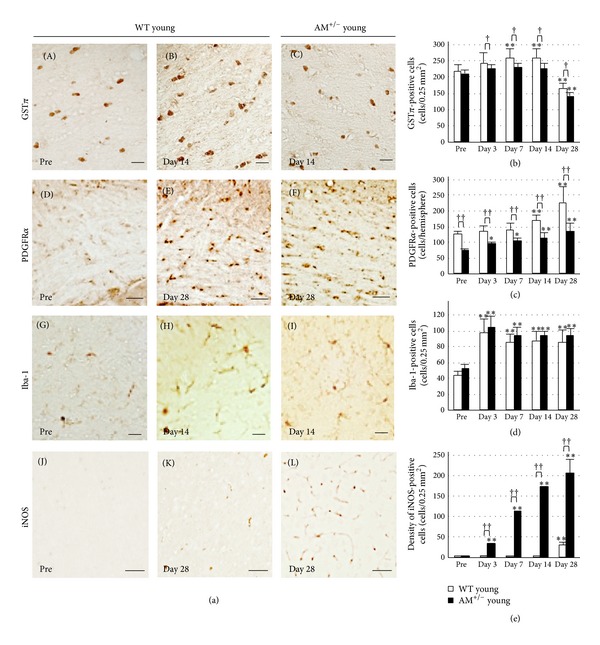
Serial changes in OLGs and OPCs after prolonged cerebral hypoperfusion in young groups. (a) Photomicrographs of GST*π*-, PDGFR*α*-, Iba-1-, and iNOS-positive cells in the corpus callosum before and after BCAS. Scale bars = 20 *μ*m (GST*π*, Iba-1), scale bars = 50 *μ*m (iNOS), and scale bars = 100 *μ*m (PDGFR*α*). ((b)–(e)) Results of quantitative analysis of the number of GST*π*- (b), PDGFR*α*- (c), Iba-1- (d), and the density of iNOS- (e) positive cells in the corpus callosum before and at days 3, 7, 14, and 28 after BCAS. Data are mean ± SEM of five mice in each group. *P < 0.05, **P < 0.001, compared with the preoperation group (pre). ^†^ *P* < 0.05, ^††^ *P* < 0.001, compared with the same time points. Pre: preoperation.

We next investigated the serial changes in oligodendrocyte and OPC counts in the corpus callosum after prolonged cerebral hypoperfusion. The number of GST*π*-stained cells (i.e., oligodendrocytes) increased gradually until 14 days after hypoperfusion (*P* < 0.05) but was lower at day 28 (*P* < 0.001) compared with the preoperation measures (Figures 3(a) and 3(b)). In contrast, the number of PDGFR*α*-stained cells (i.e., OPCs) increased marginally though significantly after the hypoperfusion (*P* < 0.05, Figures 3(a) and 3(c)). In the AM^+/−^ group, the increase in cell number was mild compared with the WT group (*P* < 0.05). Iba-1-stained cells (i.e., microglia) and the density of iNOS-positive cells were significantly higher compared with the preoperation (*P* < 0.001, Figures 3(a), 3(d), and 3(e)). Whereas the Iba-1-stained cells were comparable in AM^+/−^ and WT group (not significant, Figure 3(d)), the density of iNOS-positive cells was higher in the AM^+/−^ group compared with the WT group at all time points (*P* < 0.001, Figure 3(e), hemisphere). Importantly, white matter rarefaction and OLG and OPC activation were not observed at 7 days after BCAS but occurred in both groups with good reproducibility 14 days after BCAS. Thus, we suggest that this model may serve as a powerful tool for further investigation of the molecular pathology of WM lesions and in the design of therapeutic measures for WM lesions induced by prolonged cerebral hypoperfusion. Even after adolescence, myelin-forming mature oligodendrocytes (OLGs) in the white matter can be generated from oligodendrocyte progenitor cells (OPCs) [19, 20]. When white matter is damaged in stroke or other neurodegenerative diseases, residual OPCs rapidly proliferate, migrate to fill demyelinated areas [21, 22], and differentiate into mature OLGs to restore myelin integrity [21, 23]. An emerging concept in neuroscience emphasizes that the mechanisms of neuronal disorders involve a balance between initial injury and endogenous repair [24].

### 3.3. Changes in the Oxidative Stress between Young Groups

**Figure 4:**
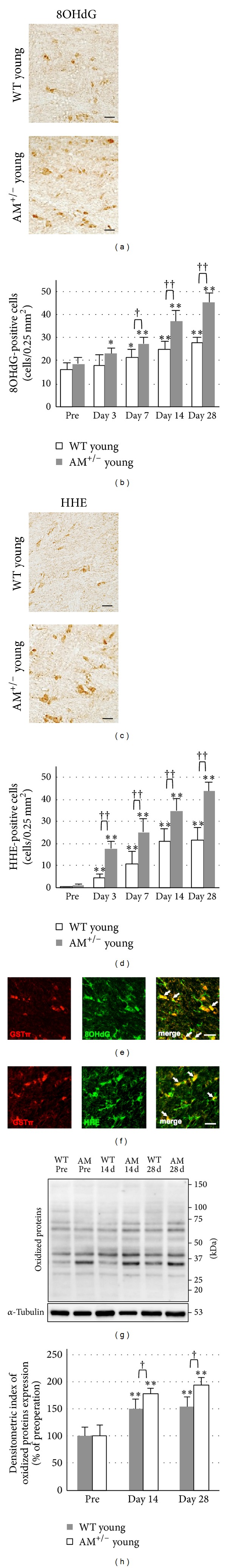
Oxidative stress after prolonged cerebral hypoperfusion in young groups. ((a)–(d)) Photomicrographs and the number of 8OHdG- ((a), (b)) and HHE- ((c), (d)) positive cells in the corpus callosum at day 28 after BCAS. Scale bars = 20 *μ*m. ((e), (f)) Double immunofluorescence staining for 8OHdG (green), HHE (green), GST*π* (red), and merged images in the corpus callosum at day 28 after reperfusion. Scale bars = 20 *μ*m. ((g), (h)) Western blotting (g) and densitometric analysis (h) of oxidized proteins. Data are mean ± SEM of five mice in each group. *P < 0.05, **P < 0.001, compared with the preoperation group (pre). ^†^ *P* < 0.05, ^††^ *P* < 0.001, compared with the same time points. Pre: preoperation.

Prolonged cerebral hypoperfusion resulted in a gradual and time-dependent increase in the number of 8OHdG- and HHE-positive cells (*P* < 0.05, Figures 4(a)–4(d)). In the AM^+/−^ group, the numbers of positive cells were higher compared with the WT group at all the time points (*P* < 0.05). We also examined the type of cells that expressed oxidative stress in the brain, using the aforementioned cell-type markers. GST*π* (marker of OLGs) was coexpressed with 8OHdG and HHE (Figures 4(e) and 4(f)). In comparison, 8OHdG- and HHE-PDGFR*α* (marker of OPCs) double-positive cells were not detected (data not shown). Western blot analysis confirmed that the density of the oxidized proteins-positive band (a marker of oxidative stress) increased in a time-dependent manner (*P* < 0.05, Figures 4(g) and 4(h)). In prolonged cerebral hypoperfusion, oxidative stress interferes with endogenous white matter repair by disrupting compensatory OPC-to-OLG differentiation [25].

A previous study used mice overexpressing circulating AM to assess the effect of AM on cerebral perfusion, cerebral angioarchitecture, oxidative stress, white matter change, cognitive function, and brain levels of cAMP, vascular endothelial growth factor, and basic fibroblast growth factor [14]. This study demonstrated contrasting results in white matter change and oxidative stress, because we used AM-knockout mice and Maki et al. [14] used AM upregulation mice. First, the number of OLGs decreased following a transient increase, but the ratio of increase was mild in the AM^+/−^ group compared with the WT group. Second, the number of OPCs gradually increased after prolonged cerebral hypoperfusion in the WT group compared with the AM^+/−^ group. Furthermore, inflammation response (iNOS) significantly increased in the AM^+/−^ group compared with the WT group. Finally, the expression of oxidative stress was detected in OLGs after prolonged cerebral hypoperfusion, with a larger increase in the AM^+/−^ group than the WT group. These results suggest that AM inactivation promotes the decrease of OLGs in a time-dependent manner after prolonged cerebral hypoperfusion, which significantly progressed white matter injury. Moreover, in white matter, lipid peroxidation products and byproducts and intermediates of aerobic metabolism and oxidative stress were increased in AM^+/−^ mice compared to WT. Oxidative stress is one cause of organ damage in a variety of pathological conditions. In particular, ischemia-reperfusion, hypertension, or diabetes mellitus increases oxidative stress and induces vascular damage, renal damage, or other organ damage [8]. AM expression is increased by oxidative stress [26, 27]. At the same time, AM inhibits oxidative stress [28]. These observations lead us to hypothesize that AM is an intrinsic antioxidant.

### 3.4. Advanced Age Increased Inflammation and Oxidative Stress

**Figure 5:**
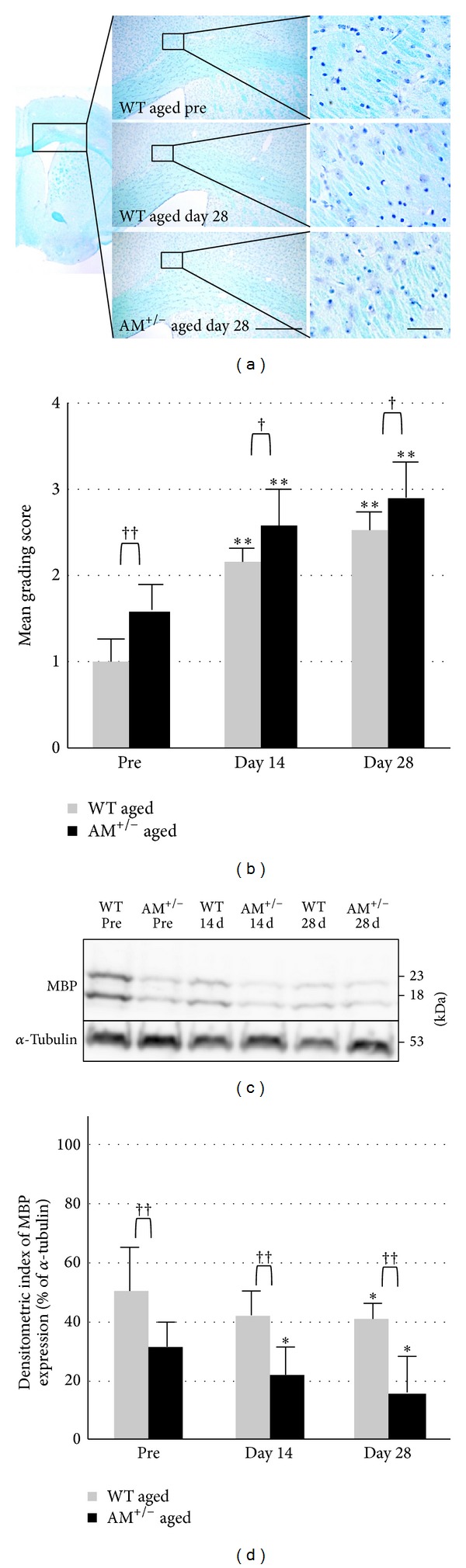
Serial changes in white matter lesion after prolonged cerebral hypoperfusion in aged groups. (a) Photomicrographs of KB staining in the corpus callosum before BCAS (pre) and at day 28 after BCAS. Scale bars = 200 *μ*m (low magnification) and 20 *μ*m (high magnification). (b) Mean grading score of KB staining. ((c), (d)) Western blotting (c) and densitometric analysis (d) of MBP. Data are mean ± SEM of five mice in each group. *P < 0.05, **P < 0.001, compared with the preoperation group (pre). ^†^ *P* < 0.05, ^††^ *P* < 0.001, compared with the same time points. Pre: preoperation.

Based on the above results, young AM^+/−^ mice showed inflammation and oxidative stress in a time-dependent manner after prolonged cerebral hypoperfusion. Previous studies indicated that long-term deficiency of AM increases insulin resistance, possibly through impaired insulin signaling in aged AM^+/−^ mice [11]. In the next step of our analysis, we compared ischemic white matter lesions between aged WT and aged AM^+/−^ mice, in which hyperglycemia had emerged. The blood glucose was comparable in young AM^+/−^ and young WT mice (109 ± 8 mg/dL versus 103 ± 6 mg/dL; not significant [NS], Figure 1(d)) but was significantly higher in aged AM^+/−^ mice than in aged WT mice (255 ± 25 mg/dL versus 127 ± 8 mg/dL, *n* = 12; *P* < 0.001, Figure 1(d)). The white matter lesions were evaluated as grade 1 in before operation and grade 2 or 3 after 14 and 28 days in aged groups (Figures 5(a) and 5(b)). The grading score was higher in the AM^+/−^ group than in the WT group at all time points (*P* < 0.05). Western blot analysis also demonstrated that the density of the MBP-positive band (18, 23 kDa) decreased in a time-dependent manner (*P* < 0.05, Figures 5(c) and 5(d)). In aging, the white matter injury was increased in the AM^+/−^ group because of high blood sugar, as previously reported [11]. With regard to cerebral ischemia/reperfusion pathophysiology, it is reported that hyperglycemia exacerbates brain injury because of poor blood flow to the ischemic penumbra, accumulation of lactate and intracellular acidosis in the ischemic brain, and enhancement of inflammatory responses [29–32].

**Figure 6:**
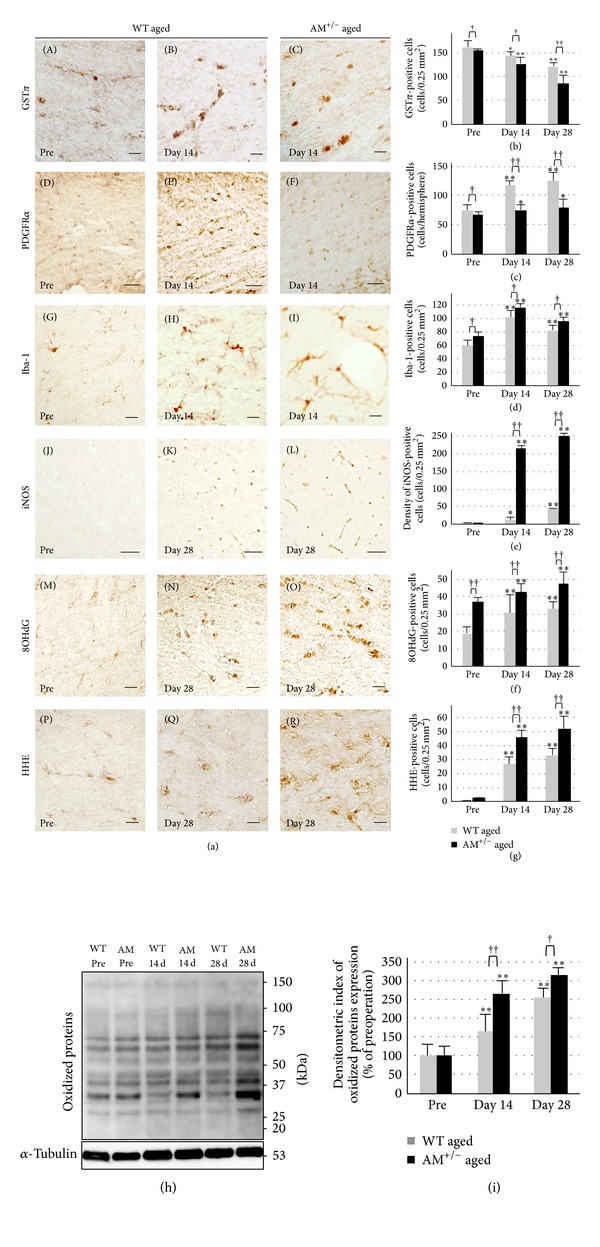
Serial changes in OLGs and OPCs after prolonged cerebral hypoperfusion between aged groups. (a) Photomicrographs of GST*π*-, PDGFR*α*-, Iba-1-, iNOS-, 8OHdG-, and HHE-positive cells in the corpus callosum at days 14 (GST*π*, PDGFR*α*, and Iba-1) and 28 (iNOS, 8OHdG, and HHE) after BCAS. Scale bars = 20 *μ*m (GST*π*, Iba-1, 8OHdG, and HHE), scale bars = 50 *μ*m (iNOS), and scale bars = 100 *μ*m (PDGFR*α*). ((b)–(g)) Results of quantitative analysis of the number of GST*π*- (b), PDGFR*α*- (c), Iba-1- (d), 8OHdG- (f), HHE- (g), and the density of iNOS- (e) positive cells in the corpus callosum before and at days 14 and 28 after BCAS. ((h), (i)) Western blotting (h) and densitometric analysis (i) of oxidized proteins. Data are mean ± SEM of five mice in each group. *P < 0.05, **P < 0.001, compared with the preoperation group (pre). ^†^ *P* < 0.05, ^††^ *P* < 0.001, compared with the same time points. Pre: preoperation.

Between aging groups, none of the GST*π*-stained cells increased after hypoperfusion; these cells decreased marginally though significantly (*P* < 0.05) compared with the preoperation (Figures 6(a) and 6(b)). This decrease was significantly higher in the AM^+/−^ group than in the WT group (Figure 6(b)). The difference in the number of PDGFR*α*-stained cells between aged groups was similar to that found between young groups (Figures 3(a), 3(c), 6(a), and 6(c)). Iba-1-stained cells and the density of iNOS-positive cells were significantly higher in aged groups compared with the young groups (Figures 3(a), 3(d), 3(e), 6(a), 6(d), and 6(e)). Moreover, advanced age resulted in a gradual and time-dependent increase in the number of 8OHdG- and HHE-positive cells (*P* < 0.05, Figures 6(a), 6(f), and 6(g)). In the AM^+/−^ group, the number of positive cells was higher compared with the WT group after hypoperfusion (*P* < 0.001). Western blot analysis confirmed that the density of the oxidized proteins-positive band increased in a time-dependent manner (*P* < 0.05, Figures 6(h) and 6(i)). Based on the above results (compared to Figures 3, 4, and 6), after prolonged cerebral hypoperfusion, the number of OPCs gradually increased in aged groups, as found in young groups, but the increased ratio was mild in aged groups compared with young groups. Therefore, the number of OLGs decreased gradually and the ratio of decrease was more severe in the AM^+/−^ group compared with the WT group. Furthermore, the inflammatory response was more severe in the AM^+/−^ group compared with the WT group. Finally, the expression of oxidative stress markers was intense at the preoperation stage in the AM^+/−^ group compared with the WT group. All factors described above progressed white matter injury in aged groups.

### 3.5. Advanced Age Decreased pCREB Expression

**Figure 7:**
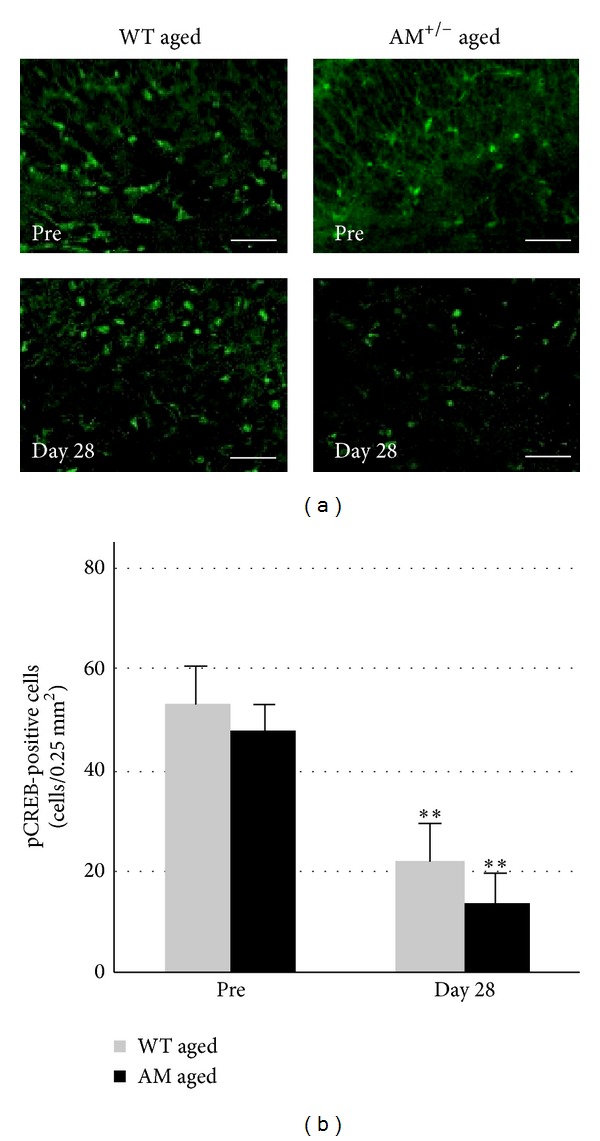
pCREB expression after prolonged cerebral hypoperfusion in aged groups. (a) Photomicrographs of pCREB staining in the corpus callosum before BCAS (pre) and at day 28. Scale bars = 20 *μ*m. (b) Numbers of pCREB-positive cells in the corpus callosum before BCAS (pre) and at day 28. Data are mean ± SEM of five mice in each group. *P* < 0.05, **P < 0.001, compared with the same time points. Pre: preoperation.

Our results indicated that white matter regions in the aged AM^+/−^ group may be vulnerable to prolonged cerebral hypoperfusion. However, the minute mechanisms are still unknown. Because CREB signaling is known to be generally important for oligodendrocyte regeneration [33], we analyzed whether differences in CREB responses may also be involved in aged-AM mice after hypoperfusion. The number of pCREB-positive cells gradually decreased in a time-dependent manner after prolonged cerebral hypoperfusion. This decrease was greater in the AM^+/−^ group compared with the WT group (*P* < 0.001, Figures 7(a) and 7(b)).

Although our study demonstrated that prolonged cerebral hypoperfusion increased oxidative stress, that inflammatory response enhanced progression of white matter damage, and that aging increased this stress and damage, there are some important caveats that need to be carefully discussed here for future studies. First, we focused only on the loss of CREB activation in aging 15-month-old white matter. However, the factors and mechanisms that may lower the CREB signaling in aged white matter were unknown. CREB activation is regulated by several growth factors, such as brain-derived neurotrophic factor, and growth factor expression decreases with age [34]. Hence, reduction of growth factor expression may cause the failure of CREB activation during stress in aged white matter. Future studies need to analyze these questions. Second, it is important to acknowledge that trying to correlate aging mouse models to the aging human brain is not straightforward. To date, most aging studies with rodents have focused on differences between young (2-3 months old) and very old (over 12–15 months old) brains [35]. However, it might be possible that the age-related decline in oligodendrogenesis would occur even before obvious declines in myelin density or cognitive function. In this study, we compared young 2-month-old mice with older 15-month-old mice. It is possible that our models may mimic old-aged humans. However, further studies are warranted to carefully track the temporal profile of these CREB-mediated mechanisms in a wider age range of mice. Third, AM is an important factor in the regulation of glycometabolism. Insulin resistance is induced by oxidative stress through a mechanism that is independent of blood pressure, and AM can act as a protective peptide against insulin resistance via its intrinsic antioxidant effect [10]. Forth, the effect of blood pressure and antioxidative/antihyperglycemic drug was not evaluated in this study. Finally, AM is multifunctional peptide (such as antiproliferative, antimigrative, anti-inflammatory, antiapoptotic, and antifibrotic effects) [1, 4]. Those effects for prolonged cerebral hypoperfusion were difficult to be excluded in this study. Future studies might answer these questions.

Long-term deficiency of AM increases oxidative stress, resulting in insulin resistance possibly through impaired insulin signaling in aged AM-deficient mice [11]. However, the precise mechanism underlying AM-induced inhibition of oxidative stress remains unknown, and further investigation is required.

## 4. Conclusion

After prolonged cerebral hypoperfusion in AM^+/−^ mice, oxidative stress and inflammatory responses enhanced progression of white matter damage, and aging and hyperglycemia increased this stress and damage. Our findings suggest that augmentation or activation of AM may suppress oxidative stress and influence the extent of ischemic white matter injury.

## Abbreviations

AM: Adrenomedullin
BCAS: Bilateral carotid artery stenosis
cAMP: Cyclic adenosine monophosphate
CBF: Cerebral blood flow
CCA: Common carotid artery
CREB: cAMP-responsive element binding protein
4-HHE: 4-Hydroxy hexenal
Iba-1: Ionized calcium-binding adapter molecule 1
OLGs: Oligodendrocytes
OPCs: Oligodendrocyte progenitor cells
PBS: Phosphate-buffered saline
pCREB: Phosphorylated cAMP-responsive element binding protein
WM: White matter
WT: Wild-type
8OHdG: 8-Hydroxy-deoxyguanosine.
